# Correspondence between multiple signaling and developmental cellular patterns: a computational perspective

**DOI:** 10.3389/fcell.2024.1310265

**Published:** 2024-07-30

**Authors:** Zahra Eidi, Najme Khorasani, Mehdi Sadeghi

**Affiliations:** ^1^ School of Biological Sciences, Institute for Research in Fundamental Sciences (IPM), Tehran, Iran; ^2^ National Institute of Genetic Engineering and Biotechnology (NIGEB), Tehran, Iran

**Keywords:** developmental pattern, signaling, cell tissue, self-organization, regenerative therapy, Turing dynamics

## Abstract

The spatial arrangement of variant phenotypes during stem cell division plays a crucial role in the self-organization of cell tissues. The patterns observed in these cellular assemblies, where multiple phenotypes vie for space and resources, are largely influenced by a mixture of different diffusible chemical signals. This complex process is carried out within a chronological framework of interplaying intracellular and intercellular events. This includes receiving external stimulants, whether secreted by other individuals or provided by the environment, interpreting these environmental signals, and incorporating the information to designate cell fate. Here, given two distinct signaling patterns generated by Turing systems, we investigated the spatial distribution of differentiating cells that use these signals as external cues for modifying the production rates. By proposing a computational map, we show that there is a correspondence between the multiple signaling and developmental cellular patterns. In other words, the model provides an appropriate prediction for the final structure of the differentiated cells in a multi-signal, multi-cell environment. Conversely, when a final snapshot of cellular patterns is given, our algorithm can partially identify the signaling patterns that influenced the formation of the cellular structure, provided that the governing dynamic of the signaling patterns is already known.

## 1 Introduction

The duality of variety and organization is among the canonical concerns in biology. During the course of development in multicellular organisms, although successive cell divisions lead to the creation of diverse cells, it does not result in colony-like accumulation of piled-up cells. Although, in principle, the genetic material of every single cell of an organism is the same, influenced by variant stimulants, they are capable of generating highly complex spatial patterns (Liu and Warmflash, 2021; Dubrulle et al., 2015; Heemskerk et al., 2019; and van Boxtel et al., 2015). A diverse range of chemical stimuli, as underlying drivers of non-genetic variations, act at multiple scales (Shahbazi et al., 2019). These stimuli play a crucial role in directing cell fate determination in stem cells at the individual cell level (Britton et al., 2021). On the other hand, collective processes such as tissue homeostasis, wound healing, angiogenesis, and tumorigenesis are intimately linked with competing environmental chemical cues (Schweisguth and Corson, 2019). Understanding the mechanisms underlying the generation and maintenance of these ordered spatial assemblies could potentially aid in the development of novel strategies for controlling tissue organization and function *in vitro* and *in vivo*.During the development of multicellular organisms, tissues are created through the spatial arrangement of differentiated cells. Although modeling the formation of a spatial arrangement from a single stem cell is complex, it becomes even more complicated in reality as tissues are formed from the spatial arrangement of cells from different stem cells. This process requires intercellular signal transmission, which affects gene expression regulation and intracellular decision-making.Internal mechanisms are responsible for generating the right proportion of different types of specialized cells, distributing them in their right position, and maintaining the organized structure in the presence of intercellular chemical signaling agents (Khorasani and Sadeghi, 2022). Cells also sense and respond to mechanical stimuli and the physical properties of their environment via induced downstream genetic regulatory networks (Valet et al., 2022; Lenne et al., 2021; Wagh et al., 2021). Several multi-stable regulatory networks play their role as the internal decision-makers of dividing cells (Khorasani and Sadeghi, 2022). This study investigates the impact of various chemical signals on the mechanism by which multiple stem cells generate intricate tissue structures and tries to provide a deeper understanding of the mechanisms behind morphological variations. In reality, the formation of intermediate structures during embryo development or the formation of a tissue consisting of cells with different phenotypes and with organization in their spatial arrangement without a previous template is a complex problem, and modeling them using the simplest possible assumptions can lead to a better comprehension of the development process in multicellular organisms.We would like to answer these questions, or, more realistically, get any enlightenment about the following: first, in the presence of variant positional cues, how can spatially organized populations give rise to and maintain large-scale inhomogeneities starting from an initially roughly homogeneous mass of intermixed stem cell populations? Second, how do individual stem cells perceive and interpret their surrounding spatial information to make decisions about their developmental pathway in response to the local concentration of these stimulants? Finally, is it possible to infer information about the specific form of the signals that created them from the final structure of cell populations?

The basis of cellular pattern formation is mounted on the interaction of the mediating nonlinear diffusive signaling components (Murray, 2001). For the spontaneous construction of patterns during development, as proposed by Turing’s classic theory, the system requires two diffusive chemical compounds: an activator compound and an inhibitor compound (Turing, 1990). The latter locally undergoes an autocatalytic reaction to generate more of itself and also activates the formation of the inhibitor compound in some way. Meanwhile, the former inhibits the formation of more activator compounds. The key element for obtaining spatial patterns is that the activator and the inhibitor components diffuse through the reaction medium at different rates. Thus, the effective ranges of their respective influences are different. Accordingly, if the inhibitor agent diffuses faster than the activator one, a stable pattern can emerge from a homogeneous background merely by the amplification of small perturbations. The patterns generally take the form of spots (and reverse spots) or stripes based on the choice of model parameters (Murray, 2001). The dynamic elaborates different possible pattern formation processes in a variety of developmental situations. The related examples span from the regeneration of hydras (Meinhardt, 2003) to animal coating patterns (Koch and Meinhardt, 1994). Wave phenomena can also generate patterns of spatiotemporal type (Cotterell et al., 2015; Eidi et al., 2021). Since the typical characteristic time of cell division is higher than that of a traveling wave, here, we exclude the formation of cellular patterns induced by spatiotemporal signaling patterns. Recently, Marcon et al. (2016) proposed a new development in classical Turing models, indicating that the essential prerequisite of varied diffusion rates for mobile signaling molecules is not essential for pattern formation. Remarkably, specific networks are capable of creating patterns using signals without the constraint of relative diffusion rates.

Here, we assume that there are two multipotent stem cells as resources of variation generation, each of which is potentially capable of constructing its own organized structure in the absence of the other. Although the cells do not directly interact, they have an intracellular signal-dependent tri-stable switch that affects their reproduction rates in response to multiple signals in the environment. We present a computational model for their internal mechanism in the presence of each other to form an organized population consisting of whole descendants. We see that signaling messengers play a significant and irreplaceable role as regulatory agents in communication between different cell types. Our results indicate that the association of variant environmental signaling messengers and intracellular decision-making switches grants a diverse range of cellular patterns. Furthermore, having the ultimate arrangement of cellular organization, one can approximately indicate the signaling patterns based on which the cellular patterns have been established, provided that the prior assumption of the pattern is given.

## 2 Materials and methods

**FIGURE 1:**
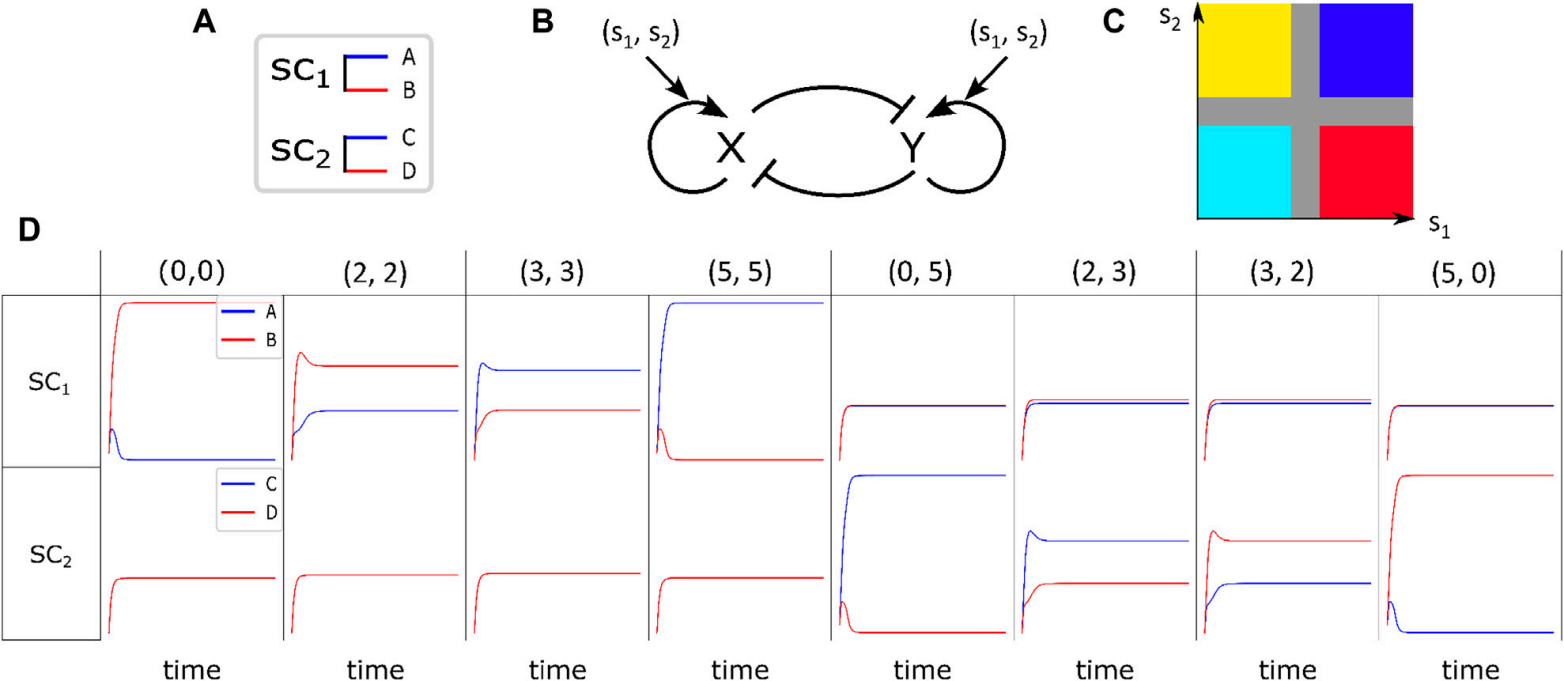
Fate determination of stem cells under the influence of environmental signaling agents. **(A)** Differentiation of $SC_{1}$ leads to either phenotype A or B , while $SC_{2}$ can differentiate into phenotype C or D depending on the amount of signaling agent exposure. **(B)** Regulation switch present in each stem cell contributes to its development toward a specific fate, which is influenced by environmental stimulant pairs $\left(s_{1},s_{2}\right)$ . **(C)** The cruciform shape in the $\left(s_{1},s_{2}\right)$ plane represents the phase field of possible developed cells. Each phenotype is color-coded, with blue representing A , cyan representing B , yellow representing C , and red representing D . The values of $s_{1}$ and $s_{2}$ have been scaled up to fall within the range of (0,5). Each quadrant in the plane corresponds to a specific phenotype. The values of $\left(s_{1},s_{2}\right)$ directly affect the determinants within the cell and, thereby, influence the outcome of cell division, as described by Equations 3, 4. The gray cross denotes a comparable concentration of signaling agents, within which randomness plays a significant role in determining cell division outcomes. **(D)** Fate of each stem cell $SC_{i}$ is influenced by a specific combination of $\left(s_{1},s_{2}\right)$ pairs. There are different pairs $\left(s_{1},s_{2}\right)$ that can affect $SC_{1}$ and leave $SC_{2}$ unaffected. For example, columns 1 to 4 can influence $SC_{1}$ and leave $SC_{2}$ neutral, while columns 5 to 8 have the opposite effect.

In this model, we consider a scenario where a plane is initially populated by two types of stem cells, $SC_{1}$ and $SC_{2}$ . These stem cells can both renew themselves and divide into their corresponding differentiated cells. When they divide into specialized cells, $SC_{1}$ can give rise to either A or B , while $SC_{2}$ can give rise to either C or D ; see Figure 1A. In this case, to simplify the computational process and maintain the essence of the scenario, we will disregard any intermediate stages and assume a direct division of stem cells into their offspring. The division outcomes of each cell are influenced by the amount of signaling agent that the mother cell receives (Khorasani et al., 2020; Khorasani and Sadeghi, 2022, Khorasani and Sadeghi, 2024). Here, the main idea is that in the absence of cellular displacement, competition between existing chemical signals in the environment plays the principal role in the pattern formation process at the population level. To model the underlying mechanism, we need to answer the following questions:

• What type of signal does the model refer to?

• How can a mixture of different signals impact the fate of an individual cell?

• What is the effect of the signals on the offspring at the population level?

The materials and methods is structured as follows: first, we introduce different possible dynamics for propagating extracellular signals in the environment, including positional information in Section 2.1.1 and reaction–diffusion dynamics in Section 2.1.2. Subsequently, in Section 2.2, we propose a regulatory switch that allows an individual cell to determine its fate influenced by the uptake of different environmental signals. Finally, in Section 2.3, we describe an algorithm for predicting the final cellular pattern of a system that is initially composed of multiple signaling agents and dividing cells.

### 2.1 Signals

Let us assume that the stem cells in a medium are exposed to spatial chemical information, we refer to them as signals, which are captured and interpreted by the cells to develop the spatial organization. There are various ways to provide spatial patterns in biology, among which, positional information and reaction–diffusion dynamics are the most prominent (Green and Sharpe, 2015).

#### 2.1.1 Positional information dynamic

**FIGURE 2:**
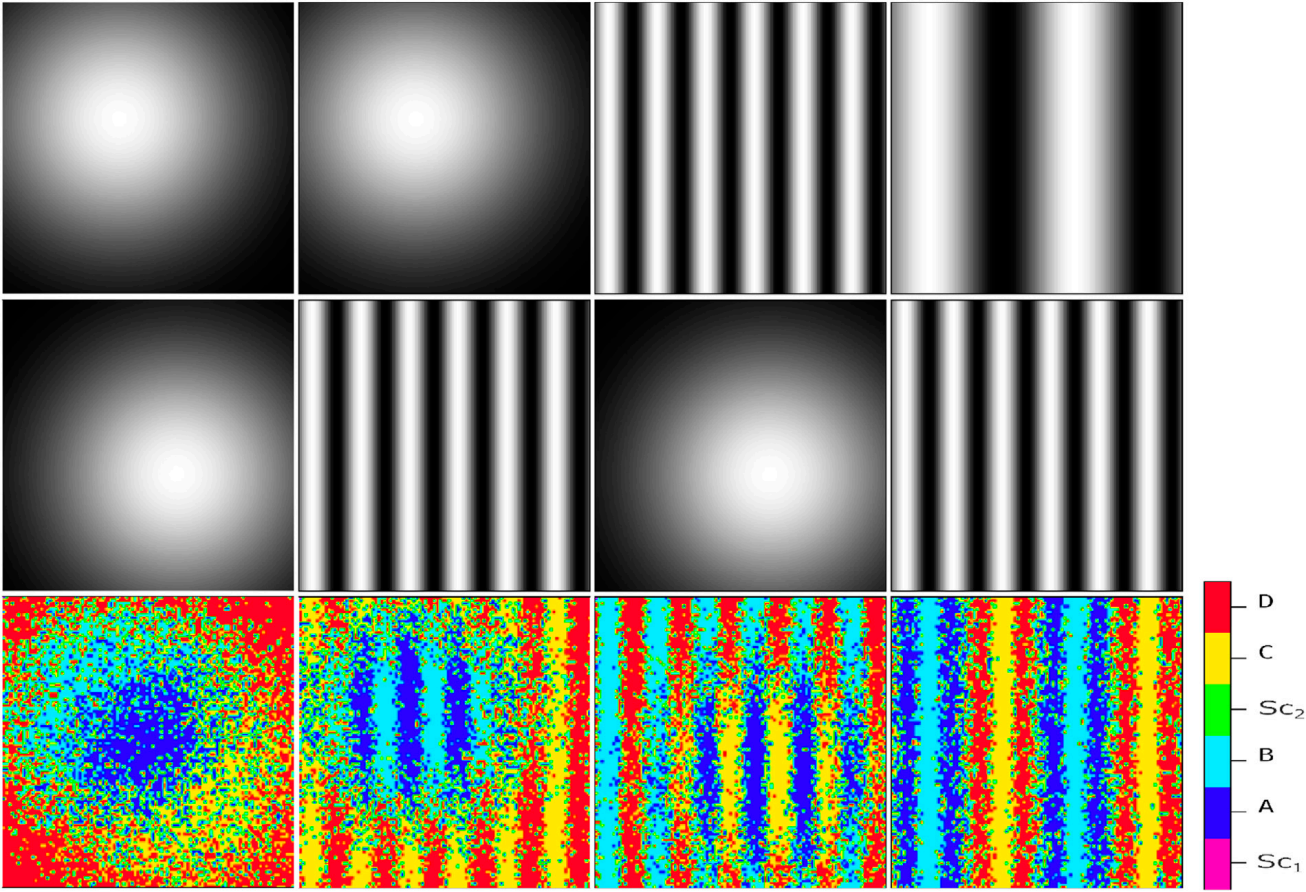
Resulting cellular pattern (third row) of a system influenced by two independent static signal profiles (first and second rows). The signal profiles consist of a Gaussian profile, described by the exponential function $\exp\left[-{\left(2\sigma^{2}\right)}^{-1}\left({\left(x-x^{*}\right)}^{2}+{\left(y-y^{*}\right)}^{2}\right)\right]$ , and a sinusoidal profile sin(kx) . The third row in each column displays the final cellular pattern resulting from stem cell division and self-renewal. Initially, stem cells are randomly distributed in an environment containing the signal profiles from the upper row. The final pattern, distinguishable by six colors, reveals specific cell types: magenta for $Sc_{1}$ , green for $Sc_{2}$ , blue for offspring A , cyan for offspring B , yellow for offspring C , and red for offspring D . The outcome depends on the comparison of signal concentrations at each point. The randomness involved in the patterns belongs to the areas where the concentration of positional signals is comparable. The first column: (top) $x^{*}=40$ , $y^{*}=30$, and σ=2, (middle) $x^{*}=60$ , $y^{*}=30$, and σ=2 (bottom) the developed pattern in consequence of the combination of its upper-head signals. The second column: (top) $x^{*}=40$ , $y^{*}=30$, and σ=2 (middle) and k=4.5 (bottom) the developed pattern in consequence of the combination of its upper-head signals. The third column (top) k=4.5, (middle) $x^{*}=40$ , $y^{*}=30$ , and σ=2 (bottom) the developed pattern in consequence of the combination of its upper-head signals. The fourth column: (top) k=1.5 (middle) and k=4.5 (bottom) the developed pattern in consequence of the combination of its upper-head signals.

Generally, positional information dynamic refers to the development of the spatial cellular organization in the embryo differentiating at specific positions based on their response to the gradient of environmental signals (Schweisguth and Corson, 2019). For example, embryonic organizer centers secrete morphogens that specify the emergence of germ layers and the establishment of the body’s axes during embryogenesis (De Santis et al., 2021). In the current study, by positional information, we mean any external chemical cues whose procedure of setting up is immaterial for us, and we merely focus on their impact on the regulation of internal switches. To illustrate the relationship between different signals, Figure 2 exemplifies the simultaneous presence of two signal profiles of Gaussian type (the first column), a Gaussian profile and a sinusoidal one (the second and third columns), and two sinusoidal with different frequencies (the fourth column). In each column, the final cellular pattern resulting from the process of cell division and self-renewal of competing stem cells is represented by the third row. Initially, the stem cells are randomly distributed in an environment that contains upper-row signals. In all cases, the final pattern can be distinguished by six different colors. The colors magenta and green represent $Sc_{1}$ and $Sc_{2}$ , respectively. The colors blue, cyan, yellow, and red are used to represent the offspring A , B , C , and D , respectively. The pattern formation process is implemented using Algorithm 1.

#### 2.1.2 Signaling through the reaction–diffusion dynamic

To generate two independent signaling agents in the medium, we consider a system that consists of two independent reaction–diffusion processes. Each process involves two interacting chemicals, namely, $s_{u}^{\left(i\right)}$ and $s_{v}^{\left(i\right)}$ , where i∈{1,2} . The spatial distribution of $s_{u}^{\left(i\right)}$ and $s_{v}^{\left(i\right)}$ is interdependent, as governed by their corresponding dynamics. Thus, there are two independent chemical variants produced by these two reaction–diffusion processes. We assume that the concentration field of $s_{u}^{\left(i\right)}$ in the medium, i∈{1,2} , defines the dynamic profile of each independent signaling agent. Moreover, $s_{u}^{\left(i\right)}$ and $s_{v}^{\left(i\right)}$ are deemed to spread over the environment with $D_{s_{u}^{\left(i\right)}}$ and $D_{s_{v}^{\left(i\right)}}$ , respectively. The governing equations for the propagation of $s_{u}^{\left(i\right)}$ and $s_{v}^{\left(i\right)}$ are as follows (Shoji et al., 2003):

$$\frac{\partial s_{u}^{\left(i\right)}}{\partial t}=\nabla^{2}s_{u}^{\left(i\right)}+\gamma f\left(s_{u}^{\left(i\right)},s_{v}^{\left(i\right)}\right),$$ (1)

$$\frac{\partial s_{v}^{\left(i\right)}}{\partial t}=d\nabla^{2}s_{v}^{\left(i\right)}+\gamma g\left(s_{u}^{\left(i\right)},s_{v}^{\left(i\right)}\right).$$ (2)

Here, by rescaling the space variable, the diffusion coefficient of $s_{u}^{\left(i\right)}$ and $s_{v}^{\left(i\right)}$ are set to 1 and d , respectively. Here, d is equal to $\frac{D_{s_{v}^{\left(i\right)}}}{D_{s_{u}^{\left(i\right)}}}$ . Thus, assuming that d≥1 , the diffusivity of $s_{v}^{\left(i\right)}$ is larger than that of $s_{u}^{\left(i\right)}$ . In addition, $f\left(s_{u}^{\left(i\right)},s_{v}^{\left(i\right)}\right)$ and $g\left(s_{u}^{\left(i\right)},s_{v}^{\left(i\right)}\right)$ are reaction kinetics of the system represented with the following terms:

$$f\left(s_{u}^{\left(i\right)},s_{v}^{\left(i\right)}\right)=As_{u}^{\left(i\right)}-s_{v}^{\left(i\right)}+C\;\;and\;\;g\left(s_{u}^{\left(i\right)},s_{v}^{\left(i\right)}\right)=Bs_{u}^{\left(i\right)}-s_{v}^{\left(i\right)}-1.$$

**FIGURE 3:**
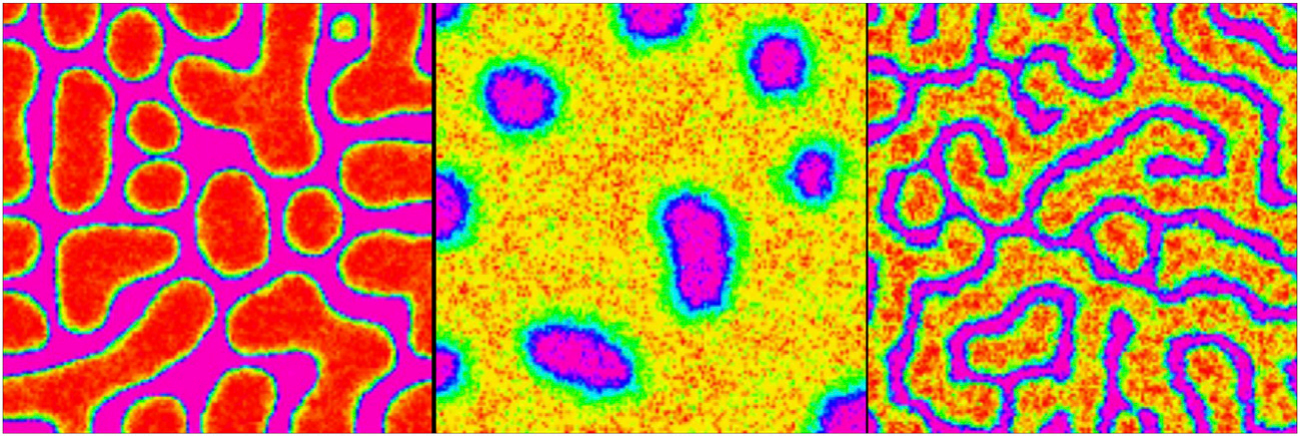
Possible signaling agent patterns $s_{i}$ $\left(s_{u}^{\left(i\right)}\right)$ following Equations 1, 2 with parameters: A=0.9 , B=1.2 , γ=10000 , and C=0.2 and lattice size h=0.01 . (Left) Spot pattern with $D_{u}=1$ and $D_{v}=20\;D_{u}$ , (middle) reverse spot pattern with $D_{u}=25$ and $D_{v}=500\;D_{u}$, and (right) stripe pattern with $D_{u}=1$ and $D_{v}=20\;D_{u}$ .

Here, A , B, and C are the controlling parameters. The kinetics also constrains the variable $s_{u}^{\left(i\right)}$ within a finite range: $0\leq s_{u}^{\left(i\right)}\leq s_{u_{\max}}^{\left(i\right)}$ . The parameter γ exhibits the relative strength of reaction kinematics. This dynamic with a reflective boundary condition can produce steady-state heterogeneous spatial patterns of chemical concentrations (Shoji et al, 2003). The diffusion process, with d≥1 , in this context, is considered the main deriving process for the heterogeneity in the system. Moreover, $s_{u_{\max}}^{\left(i\right)}$ is considered the controlling parameter, upon which the behavior of spatial patterns differs; see Figure 3. To simulate the dynamic, we implement the Gillespie method (Gillespie et al., 2007), which exhibits some degree of randomness in the simulation of chemical kinetics. The Gillespie algorithm is widely regarded as the “gold standard” for explaining the behavior of systems characterized by a limited number of determinants and driven by inherent fluctuations, all while avoiding the complexities of mathematical equations. This method generates a statistically possible solution of Equations 1, 2, for which the reaction rates are known. Defining the *propensity function* for every single reaction, including diffusion ones that are considered to be reducible to an analogous reaction, we have a measure to find out the time when the next chemical reaction takes place and determine which reaction is likely preferred by the system. The entire reactions of the system and their corresponding propensity functions are listed in Table 1. By updating the propensity functions at each step, one can track the changes in the corresponding cell-type population vector, which is induced by a single occurrence of a particular reaction. Repeating the algorithm simulates the whole behavior of the reaction–diffusion system stochastically. The complete algorithm implementation is detailed in Section 2.3. Before delving into that, it is crucial to explain how various chemical environmental signals impact the ultimate fate of an individual cell.

**TABLE 1:** Involved reactions and their corresponding propensity functions (reaction no. 1–6) generating signaling patterns and the reactions (reaction no. 7–10) involved in the production and degradation of intracellular determinants. In total, there are 20 reactions incorporated in the Gillespie algorithm, i∈{1,2} .

Reaction no.	Reaction type	Propensity function
1	Production of $s_{u}^{\left(i\right)}$	$\gamma \left(As_{u}^{\left(i\right)}+C\right)$
2	Degradation of $s_{v}^{\left(i\right)}$	$\gamma \left(s_{v}^{\left(i\right)}\right)$
3	Diffusion of $s_{u}^{\left(i\right)}$	$D_{u}/h^{2}$
4	Production of $s_{u}^{\left(i\right)}$	$\gamma \left(Bs_{u}^{\left(i\right)}\right)$
5	Degradation of $s_{v}^{\left(i\right)}$	$\gamma \left(s_{v}^{\left(i\right)}+1\right)$
6	Diffusion of $s_{v}^{\left(i\right)}$	$D_{v}/h^{2}$
7	Production of $x_{i}$	$\alpha_{x}^{\left(i\right)}\frac{x_{i}^{n}}{\beta^{n}+x^{n}}+k_{1}\frac{\beta^{n}}{\beta^{n}+y_{i}^{n}}$
8	Degradation of $x_{i}$	$\gamma_{1}x_{i}$
9	Production of $y_{i}$	$\alpha_{y}^{\left(i\right)}\frac{y_{i}^{n}}{\beta^{n}+y_{i}^{n}}+k_{2}\frac{\beta^{n}}{\beta^{n}+x_{i}^{n}}$
10	Degradation of $y_{i}$	$\gamma_{2}y_{i}$

As previously mentioned, we assume that the concentration field of $s_{u}^{\left(i\right)}$ in the medium, where i∈1,2 , represents the dynamic profile of each separate signaling agent. From this point forward, whenever we refer to $s_{i}$ , we are referring to $s_{u}^{\left(i\right)}$ .

### 2.2 Biased internal switch of determinants

Once we have identified the environmental signals that can influence the fate of stem cells, we can explore the subsequent question: how do simultaneous signals impact the destiny of a single cell?

Let us assume that within the cytoplasm of each stem cell $SC_{i}\;\left(i=1,2\right)$ , there are two interacting chemical determinants, $x_{i}$ and $y_{i}$ , where i∈{1,2} , whose values play a crucial role in determining the outcome of cell division. In this model, the interaction dynamics of these cytoplasmic determinants of the stem cell $SC_{i}\;\left(i=1,2\right)$ are controlled by a tri-stable regulatory switch. This switch controls the fate of cell division and determines whether the stem cell differentiates or self-renews. (Balázsi et al., 2011; Staff, 2017; Khorasani et al., 2020; Khorasani and Sadeghi, 2022, Khorasani and Sadeghi, (2024)). See Figure 1B.

$$\frac{\partial x_{i}}{\partial t}=\alpha_{x}^{\left(i\right)}\frac{x_{i}^{n}}{\beta^{n}+x^{n}}+k_{1}\frac{\beta^{n}}{\beta^{n}+y_{i}^{n}}-\gamma_{1}x_{i},$$ (3)

$$\frac{\partial y_{i}}{\partial t}=\alpha_{y}^{\left(i\right)}\frac{y_{i}^{n}}{\beta^{n}+y_{i}^{n}}+k_{2}\frac{\beta^{n}}{\beta^{n}+x_{i}^{n}}-\gamma_{2}y_{i}.$$ (4)

In Figure 1B, the regulatory switch is shown. It involves mutual repression of $x_{i}$ and $y_{i}$ and their degradation effects, as well as their self-activation in the form of the Hill function. In the above equations, n is the Hill coefficient, β is the synthesis rate of determinants, $\alpha_{x}^{\left(i\right)}$ and $\alpha_{y}^{\left(i\right)}$ are the self-activation rates, $k_{1}=k_{2}$ are the inhibition rates, and $\gamma_{1}=\gamma_{2}$ are the degradation rates of $x_{i}$ and $y_{i}$ , respectively.

It has been demonstrated by Khorasani et al. (2020) that in the absence of stimulant signaling chemicals, when there is only one type of stem cell and the coefficients in Equations 3, 4 are constant, there are three stable steady-states for each stem cell. These steady states correspond to three distinct cell fates: the stem cell itself and its corresponding differentiated cells. The cell’s absorption to a specific attractor is determined by the values of $x_{i}$ and $y_{i}$ , which, in turn, defines the domains of the three attractors. Building upon previous research, we aim to investigate how variant environmental chemical signals influence the concentrations of $x_{i}$ and $y_{i}$ . In this study, we consider two types of stem cells, $SC_{1}$ and $SC_{2}$ , and assume the presence of two independent signals, $s_{1}$ and $s_{2}$ , in the environment. The values of these signals evolve, and each stem cell $\left(SC_{i}\right)$ can detect the presence of both $s_{1}$ and $s_{2}$ . The cell then regulates its internal determinants based on the amount of these signals it receives, denoted as $\left(s_{1},s_{2}\right)$ . We assume that the coefficients $\alpha_{x}^{\left(i\right)}$ and $\alpha_{y}^{\left(i\right)}$ are not fixed parameters, but rather, they are influenced by environmental signals $s_{1}$ and $s_{2}$ . The behavior of $\alpha_{x}^{\left(i\right)}$ and $\alpha_{y}^{\left(i\right)}$ is governed by the following relations:

$$\alpha_{j}^{\left(1\right)}=\alpha_{0j}^{\left(1\right)}+\{\begin{array}{c} \eta \left(s_{1}\right)\eta \left(s_{2}\right)\;\;\;\;\;\;if\;\;\;\;j=x\\ \zeta \left(s_{1}\right)\zeta \left(s_{2}\right)\;\;\;\;\;\;if\;\;\;\;j=y \end{array},$$ (5)

$$\alpha_{j}^{\left(2\right)}=\alpha_{0j}^{\left(2\right)}+\left\{\begin{array}{c} \eta \left(s_{2}\right)\zeta \left(s_{1}\right)\;\;\;\;\;\;if\;\;\;\;j=x\\ \eta \left(s_{1}\right)\zeta \left(s_{2}\right)\;\;\;\;\;\;if\;\;\;\;j=y \end{array}\right.$$ (6)

Here, $\eta =\frac{s^{2}}{K_{1}^{2}+s^{2}}$ and $\zeta =\frac{K_{2}^{2}}{K_{2}^{2}+s^{2}}$ . $K_{1}$ and $K_{2}$ are the fixed parameters. We see that different concentrations of $s_{1}$ and $s_{2}$ will lead to different levels of $x_{i}$ and $y_{i}$ , which will, in turn, influence the fate of the stem cells.In this study, the parameters of Equations 3, 4 were set as follows: $\gamma_{1}=\gamma_{2}=0.38$ , β=42 , $k_{1}=k_{2}=30$ , $\alpha_{0j}^{\left(1\right)}=\alpha_{0j}^{\left(2\right)}=30$, and n=4 . Additionally, in the definition of η and ζ , both $K_{1}$ and $K_{2}$ were adjusted to equal 2.5. Finally, the parameters η and ζ were scaled up by a factor of 20. It is important to note that these parameters were determined through a trial and error process since the study was computational in nature.

### 2.3 Patterns at the population level

**FIGURE 4:**
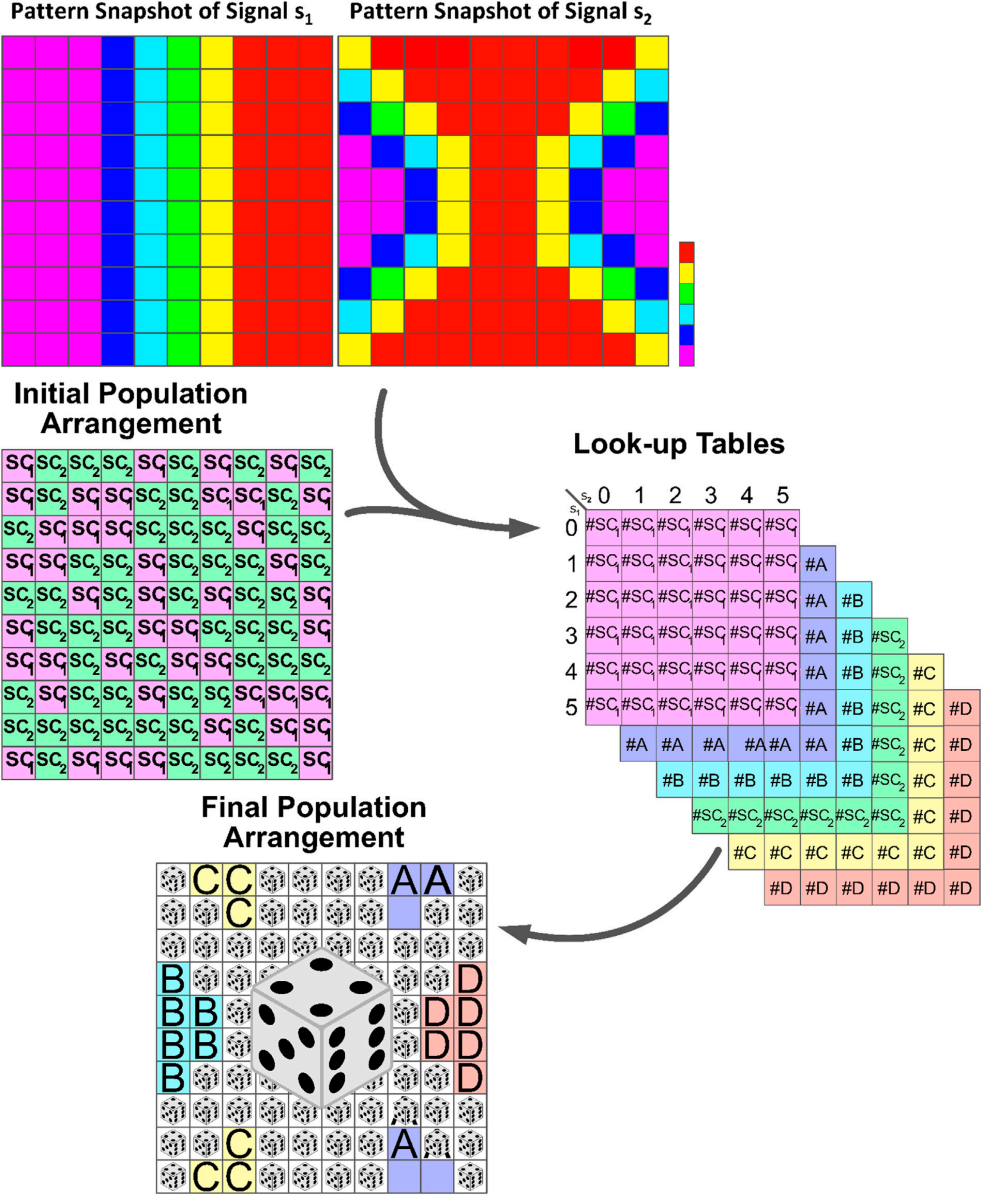
Schematic illustration of the formation of developmental patterns influenced by external signals. Stem cells respond to dynamic signals, leading to differentiation and self-renewal based on the number of determinants produced within their cytoplasm. Look-up tables summarize cell division outcomes based on the signal combination intervals. The highlighted section with dice in the lower panel represents regions with comparable signal concentrations. Here, the response of stem cells to these signals becomes unpredictable, and randomness plays an important role in determining the outcome. See Algorithm 1 for in-depth details.

In this section, we introduce a position-dependent procedure based on the Gillespie algorithm to simulate the development of differentiated cells in a population, as illustrated in Figure 4. The Gillespie algorithm is widely used for modeling systems with a small number of determinants or chemicals, taking into account inherent fluctuations. Previous studies have mainly focused on understanding the decision-making mechanism of a single type of stem cell. However, in this study, we extend our analysis to include multiple types of stem cells and various signaling stimulants in the system.

Consider a system comprising two types of stem cells, namely, $SC_{1}$ and $SC_{2}$ . These stem cells possess the capability to undergo self-renewal and differentiate into their specialized cells. The differentiation process is regulated by the presence of signaling agents $s_{1}$ and $s_{2}$ . $SC_{1}$ is capable of differentiating into A and B phenotypes, whereas $SC_{2}$ is competent to develop into C and D offspring; see Figure 1B. $s_{1}$ and $s_{2}$ independently propagate on the substrate via the reaction–diffusion dynamics of Equations 1, 2. The objective is to track the potential fate of stem cells at each location on a two-dimensional grid based on their exposure to two types of signals, $s_{1}$ and $s_{2}$ . To achieve higher accuracy, the signal levels are scaled up to a range of 0–5. For each present cell type, a 6×6 lookup table is created at the start of the simulation, where each element in the table represents the potential number of cells of the corresponding cell type after division, assuming that a specific combination of $s_{1}$ and $s_{2}$ signals $\left(s_{1},s_{2}\right)$ exists at the mother cell’s location.

The cell cycle span represents the average time interval in which each stem cell reaches the domain of one of its possible attractors: the stem cell itself or its differentiated offspring. Through trial and error, it has been determined that approximately 100 steps are necessary for the cells to reach a state of homeostasis. During this period, the values of intercellular signaling agents and intracellular determinants are updated using the Gillespie algorithm. Table 1 contains the list of reactions for these variants along with their corresponding propensity functions. Once this period is completed, the cells are ready to undergo division. At this point, we record the probable number of each possible fate based on the values of the signals and determinants. These numbers serve as the “virtual” destination of the stem cells and are recorded in their respective 6×6 look-up tables, as shown in Figure 4. The value 6 represents the resolution of the signal considered by the simulation for each cell. Consequently, the range of signal variations has been divided into six equal intervals. The selection of the element to enter the number of each probable fate in the table is directly dependent on the specific subinterval within which the values of $s_{1}$ and $s_{2}$ reside.

The process then repeats for another cell cycle duration, which is typically 100 steps. After collecting enough data in the look-up tables, for example after 1,000 steps, we can estimate the probability (Pb) of each of the six cell types being born. This is done by referring to the look-up tables and calculating the probability as the number of that particular cell type in the table divided by the total population size.

The entire process continues until the difference between two successive Pb values becomes smaller than a predefined tiny value, denoted as ϵ . This signifies that there is no significant change in the probability value and indicates the steady state of the pattern. The final pattern is constructed using the probabilities of creating each phenotype at the very last step.

To simulate the dynamic of the pattern formation through the division process provoked by the positional chemical information, we perform the following steps recurrently on a substrate of size sz=100, on which $SC_{1}$ and $SC_{2}$ have been distributed randomly (Figure 4, panel of initial population arrangement).

1. For an adequate duration, such as 100 successive steps, let the dynamic of Equations 3, 4, upon which the number of determinant agents evolves, proceed. Here, we reckon that the signaling patterns of $s_{1}$ and $s_{2}$ simultaneously evolve based on Equations 1, 2 and provoke the stem cells toward a possible destiny.
2. Follow up the “potential” destiny of the stem cell located at each grid on the plane. Allocate a 6×6 look-up table for each of the present cell types (just once at the very first iteration). We scale up the amounts of signals to the range of (0, 5). This is the variation interval of the reverse spot signals. The rows of each table represent the number of subintervals that correspond to changes in $s_{1}$ , while the columns represent the number of subintervals that correspond to changes in $s_{2}$ . Next, we need to count the number of “virtual” offspring and renewed stem cells and categorize them based on the current amount of $s_{1}$ and $s_{2}$ in each location. Then, we insert the numbers into the row and column that correspond to the subinterval where they reside in the respective cell type; see Figure 4. It is crucial to highlight that, at this point, the fate of the cells is not determined. Instead, an assessment of their potential fate can be derived by taking into account the spatiotemporal value of $\left(s_{1},s_{2}\right)$ .
3. Repeat the two previous steps 1,000 times, and record the corresponding classified data according to the above-mentioned method. In this way, one collects more data and, in consequence, the final predicted fate of the cells is closer to that of a real system.
4. Once every 1000 steps, assess the amount of $s_{1}$ and $s_{2}$ on every single grid of the main substrate and find out the corresponding number of the potential fate of the cell types on each of the six look-up tables. Then, compute the probability of the virtual emergence of each cell type simply as the number which is associated with it in the look-up table, divided by the number of the whole population. After calculating the probability of all possible outcomes of the cell-fate random variable, we compare them with the same quantities for the 1,000 steps ahead and replace their maximum difference in the d variable, which is taken as an arbitrarily large value that guarantees that we will have enough repetitions in our simulations. Repeat the above sequence of instructions until the amount of d is less than that of e=0.0025 , which is adopted as an arbitrary and constant limit for the acceptable error in our simulations.
5. Finally, substitute the initial distribution of mother cells $SC_{1}$ and $SC_{2}$ on the substrate with the final pattern of daughter cells of each type based on their come-up probability. At this stage, every single grid of the main substrate is implanted with the cell type that is more likely conformed with the influence of the signal agent pair $\left(s_{1},s_{2}\right)$ on the internal switch of determinants; see Figure 4. For a summary of the ordered process, see Algorithm 1.

In a population of stem cells, the number of dividing cells remains constant. An alternative explanation for the above algorithm can be described as follows: once the mother cells reach a state of homeostasis after 100 steps, they divide. However, the algorithm disregards the differentiated cells as the algorithm focuses on studying the internal switch of the stem cells at this stage. Thus, we assume that only the stem cell daughter cells remain at each grid point. In the next iteration, the offspring stem cells explore the phase space of $\left(x_{i},y_{i}\right)$ by updating the values of $x_{i}$ and $y_{i}$ using the Gillespie algorithm. Then, these cells are absorbed into one of the three stable states of the internal switch: the stem cell itself or its differentiated offspring. As a result, the cells divide, and the results are recorded in the look-up tables. Again, the differentiated cells are disregarded, and the process is repeated for the stem cell offspring across the entire grid. The algorithm continues until completion.

The only additional assumption in this description is that every division always yields a stem cell as its daughter cell. The difference between the two descriptions lies in the fact that the first description defines potential cell fates, while the latter assumes that the divisions are real. Both descriptions aim to gather more data, resulting in a final predicted fate of the cells that are closer to that of a real system.

## 3 Results

### 3.1 Our signal-dependent tri-stable switch works

Figure 1D illustrates the solutions of Equations 3, 4 in the presence of variant pairs of $\left(s_{1},s_{2}\right)$ . From different columns of the figure, it is evident that when the stem cells are exposed to different pairs of $\left(s_{1},s_{2}\right)$, the following fate of cell differentiation differs. The stem cells’ response to the presence of signals, which is implemented via Equations 5, 6, depends on the amount of both $s_{1}$ and $s_{2}$ . In other words, there are pair combinations of $\left(s_{1},s_{2}\right)$ that influence $SC_{1}$, while $SC_{2}$ remains neutral; *e.g.,* column 1 to 4 and vice versa (*.e.g.,* column 5–8). On the other hand, every single stem cell differentiates into one of its potential offspring based on the amount of $\left(s_{1},s_{2}\right)$ to which it has been exposed. The first row of the tabular Figure 1D displays the final course of action of $SC_{1}$ in the presence of variant combinations of $\left(s_{1},s_{2}\right)$ . As it is seen in this row, in the presence of $\left(s_{1},s_{2}\right)=\left(0,0\right)$ , B cell type is superior. The same trend is seen when $\left(s_{1},s_{2}\right)=\left(2,2\right)$ but with less difference between A and B production. In the presence of $\left(s_{1},s_{2}\right)=\left(3,3\right)$, the process is reversed, and A production becomes prior to that of B cells. When $SC_{1}$ experiences $\left(s_{1},s_{2}\right)=\left(5,5\right)$ pair signals, the A cell type becomes superior. The corresponding signal pairs of the last four columns have no impact on the preceding one of the cell types. Similarly, the second row illustrates the behavior of $SC_{2}$ in the presence of different pairs of $\left(s_{1},s_{2}\right)$ . It is seen that the first four rows have no specific influence on altering production probabilities of C and D . Although in the presence of $\left(s_{1},s_{2}\right)=\left(0,5\right)$, the production rate of C is higher, the process becomes reversed when the $\left(s_{1},s_{2}\right)$ pair reaches (3,2). When $s_{2}$ vanishes and $s_{1}$ is on its highest value*, i.e.,* 5, the production probability of D ( C ) is the maximum (minimum).

Algorithm 1 The sequential instruction to form a complex cellular pattern based on a given signaling blueprint.

ϵ←0.0025 . % a predefined small value.

d←10000 . % the difference in emerging probabilities of the six cell types between the successive steps.
 To ensure a sufficient number of iterations, the initial value of d is set as a large number.

co←0 . % a dummy counter.

sz←100 . % the number of grids on the plane.

$pb_{\mathrm{old}}\left[6\right]\left[sz\right]\left[sz\right]\leftarrow 0$.
Construct the medium and plant stem cells, $SC_{1}$ and $SC_{2}$ .
Form signal patterns s1 and s2.
**while** d≥ϵ **do**
 
co←co+1 .
 **for** i=1 *TO 100* **do**
  Update the system.
 **end**
 Let the stem cells be divided potentially, observe the offspring, and collect the data.
 **if** co%1000==0 **then**
  
$pb_{\mathrm{new}}\leftarrow$ Compute the probability of the birth of each 6 cell types based on the $s_{1}$ and $s_{2}$ values in their mother cells’ grid.
  
d← Maximum value of $\left|pb_{\mathrm{new}}-pb_{\mathrm{old}}\right|$ .
  
$pb_{\mathrm{old}}\leftarrow pb_{\mathrm{new}}$ .
 **end**
**end**
Design the corresponding medium based on the collected data

### 3.2 Individual cellular decisions lead to collective cellular patterns under the influence of combined signals

**FIGURE 5:**
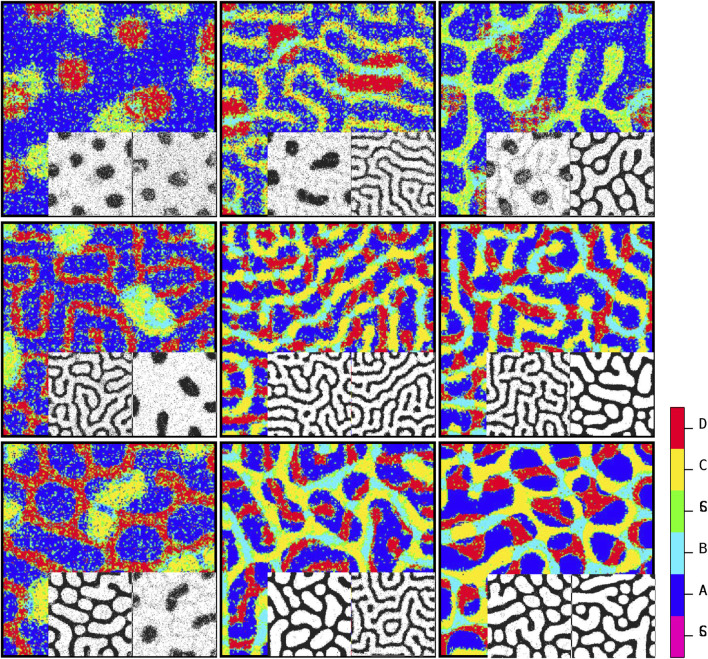
Steady-state patterns of developmental cellular arrangements in a multiple signaling field. The figures depict the patterns obtained using Algorithm 1, with each pattern governed by the dynamics of Equations 1, 2. The inline patterns, shown alongside, correspond to the signaling patterns predicted by Algorithm 2. By understanding the distribution of stimulating signals for stem cells, Algorithm 1 can determine the final cellular developmental pattern. Conversely, the inline patterns are generated according to the guidelines outlined in Algorithm 2 by analyzing the steady-state cellular pattern in each element as input. The color code reflects the cell types.

Figure 5 depicts the arrangement of the final developed cellular patterns induced as a result of variant possible combinations of signaling patterns governed by Eqs 1, 2. The color bar represents different cell types. Purple and green stand for $SC_{1}$ and $SC_{2}$ , respectively. Blue and cyan colors render phenotypes A and B, respectively, while yellow and red colors refer to C and D phenotypes, respectively. Each array of this arrangement corresponds to the combination of two members of the solution family of Eqs 1, 2, which are depicted inline in each case; see next paragraph. According to Figure 3, the solution family of these equations has three members: spot (left panel), reverse spot (middle panel), and stripe (right panel). From Figure 5, it is evident that the combination of these signaling patterns leads to a diverse collection of distinctive cellular pattern.

### 3.3 One can recognize the signal patterns from the final cellular arrangement, provided that the prior assumption of the pattern is given

Algorithm 2 The sequential instructions for determining the form of triggering signaling patterns (spot, reverse spot, or stripe) associated with the final cellular arrangement in a system of two reproducing stem cells ( $SC_{1}$ and $SC_{2}$ ) under the influence of two independent signals ( $s_{1}$ and $s_{2}$ ) in the environment. The signals are generated through a Turing process with Equations 1, 2.

sz←100 .

pMat[sz][sz]← the matrix corresponding to the population pattern, and the color code for different cell types.

s1[sz][sz]←0 . % si[sz][sz] , i∈{1,2} is the corresponding matrix of $s_{i}$ on the plane.

s2[sz][sz]←0 .
**for** i=1 *TO* sz **do**
 **for** j=1 *TO* sz **do**
  **if** pMat[i][j]==1 **then**
   
s1[sz][sz]←0.5 .
   
s2[sz][sz]←0.5 .
  **end**
  **if** pMat[i][j]==2 **then**
   
s1[sz][sz]←1 .
   
s2[sz][sz]←1 .
  **end**
  **if** pMat[i][j]==3 **then**
   
s1[sz][sz]←0 .
   
s2[sz][sz]←0 .
  **end**
  **if** pMat[i][j]==4 **then**
   
s1[sz][sz]←0.5 .
   
s2[sz][sz]←0.5 .
  **end**
  **if** pMat[i][j]==5 **then**
   
s1[sz][sz]←0 .
   
s2[sz][sz]←1 .
  **end**
  **if** pMat[i][j]==6 **then**
   
s1[sz][sz]←1 .
   
s2[sz][sz]←0 .
  **end**
 **end**
**end**

Figure 5 depicts the ultimate configurations of cellular arrangements resulting from different combinations of signaling patterns generated by Eqs 1, 2. The two corresponding acquired signaling patterns are displayed at the bottom right of each array. The key point here is that there is a dual relationship between the signal distribution and cell growth pattern. By understanding the distribution of signals that stimulate stem cells, algorithm 1 can be utilized to ascertain the final cell growth pattern. Conversely, by knowing the specific types of signals present, algorithm 2 can be employed to determine the parameters associated with the signal pattern based on the final cellular arrangement. In other words, if we are provided with a snapshot of the steady state of a developed cellular pattern and we assume that this pattern is influenced by two independent signals (s1 and s2) generated through a Turing process with Eqs 1, 2, algorithm 2 can predict the shape of each signal (spot, reverse spot, or stripe) based on the observed final cellular pattern. Recognition of signal patterns is a directional process. Algorithm 2: first, it is necessary to consider two blank planes, each of which is in accord with one of the signals to project its corresponding pattern onto it. Next, we go through every single pixel of the cellular pattern. Then, based on the color of the pixel, we map the projection of this color onto the signal planes. Let us assume that the color of a pixel is blue, meaning that this pixel is occupied with a cell of phenotype A . According to the relations (Eqs 5, 6) as well as Figure 4, this implies that at this spot, the concentration of both signals is approximately at its own summit. As a result, the projection of every blue pixel of the cellular pattern on both signal planes is a white point. Similarly, the cyan color in the cellular pattern corresponds to the B phenotype, whose occurrence is highly probable when the concentration of both signals is low. Accordingly, the map of each cyan pixel matches a corresponding black color on both signal planes. Likewise, the yellow (red) color represents the C phenotype ( D phenotype), whose production rate is high when the concentration of $s_{1}$ is low (high), while that of $s_{2}$ is high (low). As a consequence, the projection of each yellow (red) pixel onto the corresponding point on the first signal plane is white (black), while its projection onto the similar point on the second signal plane is black (white). For a summary of the ordered process, see Algorithm 2.

## 4 Discussion

The positional stimuli have been emerging as key regulators of transcription and gene expression in diverse physiological contexts (Rulands et al., 2018). These environmental drivers engage in the phenotypic diversity and proliferation/differentiation balance of stem cells (Balázsi et al., 2011; Rulands and Simons, 2016; Blake et al., 2006). The regulation process of non-genetic diversity involves the interplay of intracellular and intercellular components to interpret positional cues (Çağatay et al., 2009; Acar et al., 2008). In a competing arena in which various chemical stimulants vie for affecting a cell’s fate more, the process demands more robust and complex mechanisms. In order to specify and extend their offspring territory, the stem cells utilize a signaling process to communicate and collaborate with each other. This process ends in collective self-organized forms on length scales that are much larger than those of the individual units Chhabra et al. (2019).

In this study, first, we investigated the impact of multiple passive external signals on intracellular switches of a single stem cell. This provides us with a direct inspection of the connection between intracellular and extracellular dynamics. By mapping the environmental signaling patterns to the probability of the emergence of differentiated cell types, this model is capable of capturing any desired complex pattern, whether passive or active. The sort of models that recapitulates signaling dynamics and predicts cell fate patterning upon chemical perturbations precedingly has been investigated in non-competitive environments (Khorasani and Sadeghi, 2022; Khorasani et al., 2020; Sharifi-Zarchi et al., 2015; Chambers et al., 2007; Kalmar et al., 2009; Chen et al., 2010; Bergsmedh et al., 2011). Here, we focused on the behavior of each cell in the interaction with multiple signals. Figure 2 illustrates the resulting phenotypic cellular patterns of different combinations of two typical signal profiles of Gaussian and sinusoidal blueprints.

The environmental signals influence the fate of each stem cell, SCi (i = 1,2), by means of biasing the regulation of our tri-stable switch; see Equations 3, 4. Based on the definition of $\alpha_{j}^{\left(1\right)}$ and $\alpha_{j}^{\left(2\right)}$ in Equations 5, 6, it is evident that the pairs of $\left(s_{1},s_{2}\right)$ are relevant in controlling the decisions of this switch. This definition is advantageous in various aspects: first, it directs each stem cell’s fate to the symmetric phase space of Figure 1C, where each of the patches correspond to one of the resulting phenotypes and there is no dominant domain between them. In addition, the representative patches are far enough apart to lead to distinctive outcomes in the occurring cellular pattern field. The narrow cruciform band, *i.e.,* the gray area in Figure 1C between these four patches, is where the fate of each cell is determined stochastically. From Figure 1D, it is evident that the regulatory switch plays either an active role or a neutral one based on the amount of existing signals $\left(s_{1},s_{2}\right)$ in each point, *i.e.,* combinations of $\left(s_{1},s_{2}\right)$, which effectively lead to offspring A or B from $SC_{1}$, have nothing to do with $SC_{2}$ and *vice versa*. In consequence, there is a smooth transition from left to right in each row of Figure 1D.

After investigating the impact of static environmental stimulants on the internal switch, we dealt with the active signaling between the sources that produce variant phenotypes. We took advantage of confined Turing models for two different signals secreted from each of stem cells (Shoji et al., 2003). The dynamic includes linear reaction terms and additional constraints that confine the two variables within a finite range. The resulting patterns of this dynamic are either stationary striped patterns or spotted patterns. The second pattern, in turn, consists of two forms: spotted and reverse spotted patterns. Here, the tuning parameter upon which the pattern type is specified is the maximum concentration of the activator $s_{u}^{\left(i\right)}$ , where i∈1,2 (Shoji et al., 2003); see Figure 3. Based on this prior dynamic, nine distinct mutual patterns are generated by the two signals $s_{u}^{\left(1\right)}$ and $s_{u}^{\left(2\right)}$ .

Stochasticity has been proven to be a non-genetic diversifying resource of variation in nature (Delbrück, 1940; McEntire et al., 2021; Acar et al., 2008; Kepler and Elston, 2001; Wu and Tzanakakis, 2012; Perez-Carrasco et al., 2016). It has been shown that controlled amount of randomness ends in phenotypic variation and, as a result, population heterogeneity (Losick and Desplan, 2008; Greulich and Simons, 2016; Khorasani et al., 2020). In this study, to reflect the non-deterministic portion of the signaling system, we implemented the Gillespie algorithm (Gillespie et al., 2007) by stepping in time to successive molecular reaction events according to the premises of the model of Shoji et al. (2003); see Equations 1, 2. Another aspect of incorporating randomness in our reductionist insight is simulating the emergence of every cell type in the look-up table based on the calculation of its corresponding probability; see Figure 4. Stochastic algorithms generally provide the chance to explore multiple solutions and potentially uncover a better one compared to a deterministic method, which may get stuck in a local minimum (Gillespie et al., 2007). Additionally, these algorithms can be easily tailored to different problems and constraints, making them adaptable for solving more complex issues. By utilizing stochastic methods, we can account for the inherent randomness and fluctuations present in natural systems. This strategy allows for controlled noise to be introduced into the system. As long as the level of randomness is controllable, the system’s behavior remains predictable, and the resultant patterns are statistically reproducible.

In our algorithm, we evaluate the similarity between cellular patterns exposed to different pairs of signaling patterns by comparing them numerically to a cellular pattern constructed through a deterministic process while being exposed to the same pair of signaling patterns. This measure of similarity serves as an indicator of the reproducibility of cellular patterns using the algorithm proposed. To accomplish this, we first create two new 100×100 matrices, each corresponding to one of the signaling patterns. The size of the matrices corresponds to the plane on which the signals are distributed, with each element indicating the quantity of a specific signal at each grid location. The elements of these newly constructed matrices are either zero or the maximum value of that signal based on the corresponding elements in the original signaling matrices. If the original signal matrix element is less than half of its maximum number, the element in the new matrix is set to zero. If the element is greater than or equal to half of its maximum number, it is replaced by the maximum value of that signal. For example, when two reverse-spot type signaling patterns are distributed in the medium, each with a maximum value of 5, there are four possible pairs of extreme signals: (0,0),(0,5),(5,0), and (5,5). From Figure 1, it is evident that these pairs of signals lead to the emergence of A , C , D , and B types of cells, respectively.

**FIGURE 6:**
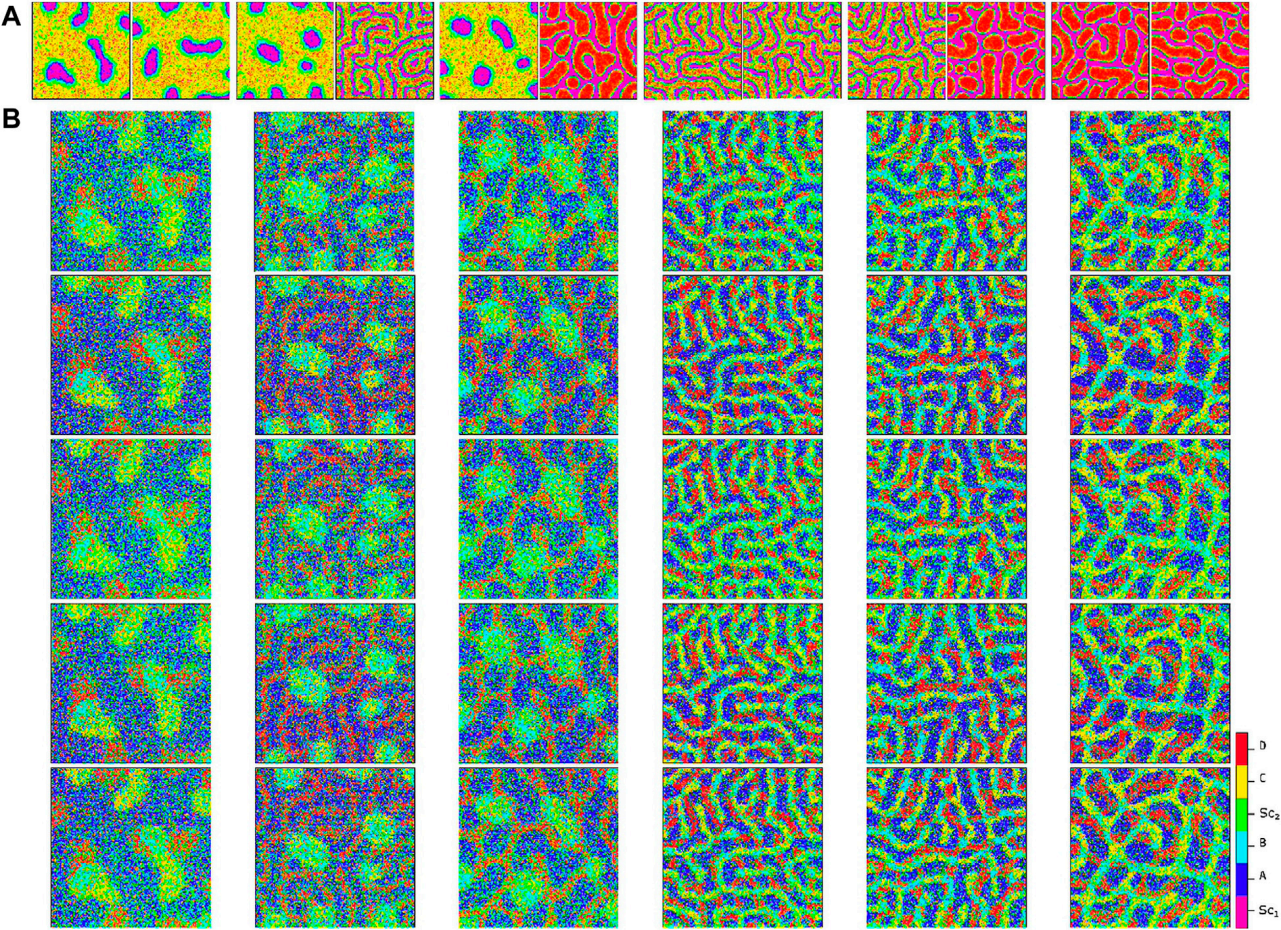
Comparison of cellular patterns for multiple pairs of signaling patterns simulated using the proposed algorithm. Panel **(A)** illustrates the signaling patterns pairs at the top of each column, while Panel **(B)** shows five independent simulated cellular patterns for each pair. The apparent similarity of the cellular patterns demonstrates the reproducibility of the method. Refer to Figure 7 for an analytical measure of the pattern similarity.

**FIGURE 7:**
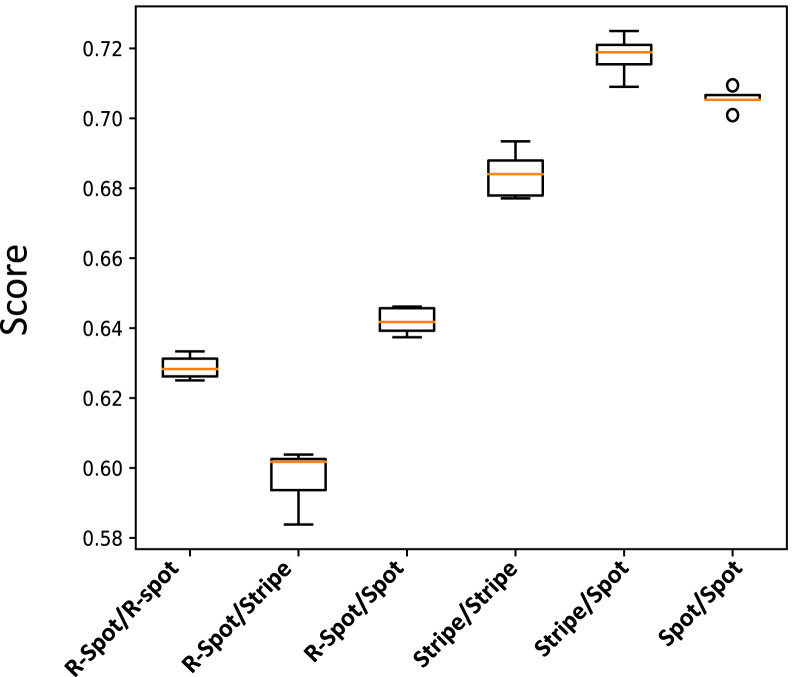
Box plot illustrating the normalized score values for the cellular patterns depicted in Figure 6. The score value measures the similarity of each pattern to a reference deterministic pattern created from their extreme signaling value pairs.

By using these extreme signaling patterns, we can determine the fate of each stem cell in the medium and create a deterministic cellular pattern accordingly. We now have a reference pattern to assess the reproducibility of our algorithm and measure the resemblance of different patterns exposed to similar pairs of signaling patterns. Figure 6 illustrates five different realizations for various pairs of signaling patterns. The signaling patterns are shown above each column, and the resulting cellular population realizations are displayed below them in each column. We can compare each realization with its corresponding deterministic pattern, pixel by pixel. If the cell types in a pixel are identical, we assign a score of +1 for the resemblance of the pattern to the deterministic reference pattern. The normalized score function, which quantifies the resemblance, is the sum of all these +1’s divided by the population size. Figure 7 shows the box plot of the score variable for the cellular patterns in Figure 6 with respect to the different signaling pattern pairs. It is observed that for all the pairs, the median of the scores is above 0.6. We see that the synthesis of the signaling arrangement with the switch in the presence of controlled noise creates rich and highly reproducible organizations of differentiated cells. Figure 5 depicts the resulting patterns of the differentiated cells that have been exposed to various combinations of active signaling lay-outs of Figure 3. The procedure we dealt with in this study is one of the various known roots to construct an organized arrangement of cells. Mobility of cells (Gallagher et al., 2022), modulation of the physical and geometrical environment (Valet et al., 2022), and priming with chemical signals (Shahbazi et al., 2019) are among other intrinsic capacities of stem cells to make patterns. In practice, a combination of all these methods is incorporated to form an organization (Omid-Shafiei et al., 2023). Nevertheless, it is seen that solely following chemical environmental cues leads to the production of a rich and wide range of patterns.

In conclusion, this study demonstrates that the signal-dependent tri-stable switch can serve as a useful tool to bridge intracellular dynamics with intercellular structures. In this scenario, although the stem cells do not directly interact with each other, their reproduction rates are influenced by external signals in their environment through the switch mechanism within each cell. By studying individual cellular decisions and the influence of multiple signals, we observe how complex cellular patterns emerge. Although the algorithm utilized in this study simplifies certain aspects, such as considering the dynamic environment during cell division, apoptosis, and cell movement, the presented systematic approach allows for the simulation of complex cellular organizations based on fundamental biophysical processes, resulting in reproducible outcomes. Moreover, for any given complex cellular pattern, for which merely the prior class of signal patterns is known, the provided method closely concludes the signaling profile that sets off the cellular pattern. Overall, these findings highlight the potential of using signal-dependent switches for better comprehension and regulation of cellular behaviors in diverse scenarios.

## Data Availability

The original contributions presented in the study are included in the article/Supplementary Material; further inquiries can be directed to the corresponding author.
